# MRI based radiomics nomogram for predict recurrence of non functioning pituitary macroadenomas post surgery

**DOI:** 10.1038/s41598-025-89907-z

**Published:** 2025-04-14

**Authors:** Ji-ping Zhao, Xue-jun Liu, Hao-zhi Lin, Chun-xiao Cui, Ying-jie Yue, Song Gao

**Affiliations:** 1 Department of Radiology, The Affiliated Hospital of Qingdao University, Qingdao, China; 2 Department of Stomatology, The Affiliated Hospital of Qingdao University, Qingdao, China

**Keywords:** Pituitary, Adenoma, MRI, Radiomics, Recurrence, Cancer, Diseases

## Abstract

Objective: To establish and validate a comprehensive predictive model combining clinical data and radiomics features to improve the accuracy of predicting recurrence within five years after surgery in patients with non-functioning pituitary macroadenomas (NFMA). Methods: This retrospective study included 292 NFMA patients who underwent surgery between January 2012 and January 2018, with an additional 123 patients as an external test set. Clinical, pathological, and conventional imaging features were collected and analyzed using univariate and multivariate logistic regression to identify independent risk factors for postoperative recurrence. Radiomic features were extracted from preoperative T1-weighted (T1WI), T2-weighted (T2WI), and T1-enhanced images using 3D Slicer software. A radiomics prediction model was developed, and a combined model integrating clinical and radiomics features was established. The predictive performance of the models was evaluated using receiver operating characteristic (ROC) curves, calibration curves, and decision curve analysis (DCA). Results: The clinical model (Cli-score), radiomics model (Rad-score) and combined model were developed. The diagnostic performance of the clinical model in the external test set, showed an AUC of 0.757 (95%CI: 0.671–0.830), with SEN, SPE, and ACC of 82.5%, 59.04%, and 71.54%, respectively. The diagnostic performance of the radiomics model in the external test set showed an AUC of 0.835 (95% CI: 0.757–0.896), with 80%, 79.52% and 63.41% for SEN, SPE and ACC%, respectively. The diagnostic performance of the combined model in the external test set showed an AUC of 0.863 (95% CI: 0.790–0.919), with SEN, SPE, and ACC of 80%, 81.93%, and 68.30%, respectively. The calibration curve indicated good predictive performance, and DCA confirmed the high clinical utility of the combined model. Conclusion: The combined model provides a more accurate prediction of NFMA recurrence. This model can guide postoperative follow-up strategies and aid in early initiation of adjuvant therapy for high-risk patients.

## Introduction

Pituitary adenomas are common intracranial tumors, accounting for approximately 10–15% of all intracranial tumors^1^. Pituitary tumors can be classified based on various criteria such as size, immunohistochemistry, invasiveness, hormone secretion, and clinical presentation^2,3^. Clinically, pituitary tumors are classified into functioning and non-functioning pituitary adenomas^4^. Non-functioning pituitary macroadenomas (NFMA), defined as non-functioning pituitary adenomas with a diameter greater than 10 mm, are a common type of pituitary tumors^5,6^.

Despite most NFMA being diagnosed as benign adenomas, some NFMA may experience early progression/recurrence after surgical removal^7^. Recurrence occurs in 12-58% of patients with residual adenomas^8,9^. Although postoperative adjuvant radiotherapy can reduce the recurrence of NFMA after surgery, this approach may lead to irreversible pituitary dysfunction and other long-term complications^10^. Therefore, studying predictive factors for postoperative recurrence in NFMA patients is of great significance. Previous studies have shown that many clinical and pathological prognostic indicators may predict the recurrence of pituitary adenomas, including immunohistochemical characteristics, tumor invasiveness, gene expression, and proliferation markers^11^. Traditional MRI features, such as invasion of the cavernous sinus, extrasellar extension, and the presence of tumor apoplexy, have been reported as important imaging features associated with recurrence of NFMA^12,13^. However, these parameters are mostly qualitative and subjective, and there are differences between observers. Radiomics is an analytical form that quantitatively extracts imaging features from medical data^14^, with higher information utilization and its quantitative analytical methods making the results more objective and fair. So far, radiomics studies of pituitary adenomas have mainly focused on two aspects: preoperative assessment and subtype classification^15^. Some researchers have used radiomics to explore the possibility of pituitary adenoma recurrence. However, most of these studies have small sample sizes and only involve single-sequence radiomics research^16,17^. We aim to establish a comprehensive classification model that combines independent clinical, conventional imaging, pathological, and preoperative radiomics features to predict recurrence within 5 years after NFMA surgery.

## Materials and methods

### General information

**Fig. 1:**
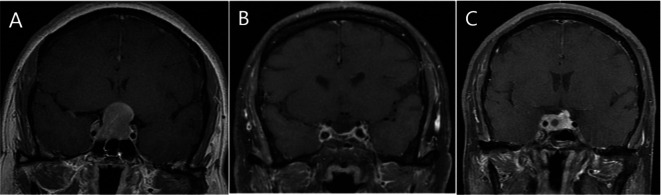
Preoperative T1-enhanced MRI image of the patient (A) shows a lesion in the saddle area, with the surgical pathology indicating a pituitary adenoma. The 4-month postoperative re-examination MRI of the saddle area (B) shows no obvious tumor residue, while the 3-year postoperative re-examination MRI (C) shows a mass in the saddle area, suggesting tumor recurrence.

From January 2012 to January 2018, a total of 292 NFMA patients who underwent surgery at our hospital were included in this study. Additionally, 123 NFMA patients treated at Shandong Provincial Hospital from June 2012 to January 2018 were collected as an external test set. All patients were followed up postoperatively through MRI examinations. The follow-up period for patients without recurrence was no less than 5 years. For those with recurrence, the discovery of tumor recurrence was considered the endpoint of follow-up. Based on clinical symptoms, MRI findings, and secondary surgical pathology, the cases were divided into recurrence and non-recurrence groups. The appearance of a new tumor mass in the sellar region or an increase in residual tumor was considered tumor recurrence (Fig. 1). A total of 292 patients with NFMA from our hospital were included in this study, who were randomly divided into a training set and a validation set in a 7:3 ratio. The training set consisted of 204 cases, with 84 patients experiencing recurrence within 5 years after surgery and 120 patients without recurrence. The validation set had 88 cases, with 36 patients experiencing recurrence within 5 years after surgery and 52 patients without recurrence. The external test set included 123 patients, with 40 patients experiencing recurrence within 5 years after surgery and 83 patients without recurrence.

### Inspection method

All patients underwent preoperative MR plain and enhanced scans. The scanning was performed using GE Signa 1.5T and 3.0T MRI machines with a 32-channel head coil. The scanning sequences and parameters were as follows:

T1WI (FSE sequence, TR: 330-430ms, TE: 15-18ms, in the coronal and sagittal planes, slice thickness: 3.0 mm, gap: 0.5 mm, NEX: 2).

T2WI (fast FSE sequence, TR: 2380-2580ms, TE: 60-108ms, in the coronal plane, slice thickness: 3.0 mm, gap: 0.5 mm, NEX: 2).

T1 enhanced fat suppression (FSE sequence, TR: 423-680ms, TE: 10-17ms, in the coronal, axial, and sagittal planes, slice thickness: 3.0 mm, gap: 0.5 mm, NEX: 2), with intravenous injection of 0.1mmol/kg body weight of gadopentetate dimeglumine (Gd-DTPA) contrast agent.

Patients in external test set underwent preoperative MR plain and enhanced scans. The scanning was performed using GE Signa 1.5T and 3.0T MRI machines with a 32-channel head coil. The scanning sequences and parameters were as follows:

T1WI (FSE sequence, TR: 340-420ms, TE: 10-18ms, in the coronal and sagittal planes, slice thickness: 3.0 mm, gap: 0.5 mm, NEX: 2).

T2WI (fast FSE sequence, TR: 2000-2680ms, TE: 40-102ms, in the coronal plane, slice thickness: 3.0 mm, gap: 0.5 mm, NEX: 2).

T1 enhanced fat suppression (FSE sequence, TR: 412-703ms, TE: 10-17ms, in the coronal, axial, and sagittal planes, slice thickness: 3.0 mm, gap: 0.5 mm, NEX: 2), with intravenous injection of 0.1mmol/kg body weight of gadopentetate dimeglumine (Gd-DTPA) contrast agent.

### Establishment of clinical prediction model

In the training set, the basic clinical information of the patients (age, gender, clinical symptoms), surgical and postoperative pathological information (including the extent of tumor resection, tumor consistency, tumor hormone type, Ki-67 proliferation index), and conventional radiological features (including tumor morphology, involved site, maximum diameter of the tumor, volume of the tumor, intratumoral hemorrhage, cystic changes within the tumor, uniformity of tumor signals, Knosp grading of the tumor, signal intensity ratio of solid part on T1WI, T2WI, and T1-weighted enhanced images) were collected. Through univariate and multivariate logistic regression analysis, with the Akaike Information Criterion (AIC) as the basis, independent risk factors associated with postoperative recurrence of non-functional pituitary adenomas were identified. Additionally, the odds ratios (OR) and regression coefficients of different risk factors were calculated. A clinical prediction model was ultimately established, and the clinical factor score (Cli-score) was calculated. The calculation method involved multiplying the clinical, routine MR, and pathological feature values by their respective coefficients, summing the products, and adding a regularization term.

### Establishment of radiomics prediction model

A radiologist with over 5 years of experience used 3D Slicer software to segment the preoperative axial T1WI, T2WI, and T1-enhanced images of the patients. The radiologist was trained to follow a standardized protocol to ensure consistency. The protocol involved identifying the boundaries of the pituitary macroadenoma based on specific anatomical landmarks and signal characteristics on the MRI scans. The radiologist was blinded to patient outcomes to avoid bias. Initial segmentations were reviewed by a consensus panel to resolve discrepancies. Random samples were reviewed by an independent expert to ensure quality and consistency.To minimize the impact of segmentation errors, MRI images of 20 NFMA patients were randomly selected and segmented by another radiologist with over 10 years of experience. The inter- and intra-class correlation coefficients (ICC) were calculated to assess the consistency and repeatability of segmentation results among different observers.

For the segmented tumor ROIs, radiomic features were extracted using the 3D Slicer Radiomics plugin. Prior to feature extraction, the images were standardized. The bin width was set to 25 for grayscale discretization; all images and corresponding ROI masks were resampled to a resolution of 1 mm×1 mm×1 mm. A total of 1,130 radiomic features were extracted for each sequence, including:

First-order features (*n* = 18) Mean: Reflects the average gray level value within the tumor, related to the overall metabolism of the tumor. Variance: Reflects the degree of variation in gray level values within the tumor, related to the heterogeneity of the tumor. Skewness: Reflects the asymmetry of the gray level value distribution, related to the non-uniformity of the tumor. Kurtosis: Reflects the sharpness of the gray level value distribution, related to the distribution of extreme values in the tumor.

Texture features (*n* = 75) Gray Level Co-occurrence Matrix (GLCM, 24 features): Describes the spatial relationship and texture information between pixels in an image, reflecting the texture heterogeneity within the tumor, and related to the invasiveness and proliferative ability of the tumor. Gray Level Size Zone Matrix (GLSZM, 16 features): Describes the characteristics of homogeneous regions, reflecting the distribution of regional sizes within the tumor, and related to the growth pattern and invasiveness of the tumor. Gray Level Dependence Matrix (GLDM, 14 features): Describes the dependence relationship between pixels with identical gray levels in an image, reflecting the gray-level dependency within the tumor, and related to the microstructure of the tumor. Gray Level Run Length Matrix (GLRLM, 16 features): Describes the texture continuity in an image, reflecting the texture continuity within the tumor, and related to the invasiveness of the tumor. Neighborhood Gray Tone Difference Matrix (NGTDM, 5 features): Describes differences between each voxel and its neighboring voxels, reflecting the gray-level differences within the tumor, and related to the microstructure and invasiveness of the tumor.

Shape and size features (*n* = 14) Volume: Reflects the overall volume size of the tumor, related to the growth stage of the tumor. Surface Area: Reflects the surface area size of the tumor, related to the invasiveness of the tumor. Sphericity: Reflects whether the shape of the tumor is close to spherical, related to the growth pattern of the tumor.

All first-order and texture features were transformed using filters to extract the Laplacian of Gaussian (LoG) features, totaling 279. The ROI was decomposed into low-frequency components (L) or high-frequency components (H) in the x, y, and z directions, resulting in 8 wavelet features (LLL, LLH, LHL, LHH, HLL, HLH, HHL, HHH), totaling 744. Altogether, 3,390 radiomic features were extracted from the three sequences.

ICC was calculated using the radiomic features from the 20 cases that were segmented by different experimenters. Features with an ICC value greater than 0.75 were selected for the next step of analysis. Single-variable selection of features was conducted using variance analysis, followed by dimensionality reduction and feature selection using the least absolute shrinkage and selection operator (LASSO) with 10-fold cross-validation. Features were further screened using a logistic regression model, with AIC serving as the stopping rule for backward stepwise selection.

A predictive model was established using multiple logistic regression, and the radiomics score (Rad-score) was calculated. The calculation method was the sum of the radiomic feature values multiplied by their respective coefficients, plus a regularization term.

### Establishment of the combined predictive model

Incorporate the selected clinical, conventional imaging, pathological features, and Rad-score into a multivariable logistic regression analysis to construct a combined predictive model. Sum the scores of different features to obtain the prediction score of the combined model (Combined score). Visualize the results more intuitively by plotting a nomogram. Use a calibration curve to assess the similarity between the predicted and actual postoperative tumor recurrence.

### Validation and prediction performance evaluation of different predictive models

Plot the receiver operating characteristic (ROC) curves for different models, calculate the sensitivity (SEN), specificity (SPE), accuracy (ACC), and the area under the ROC curve (AUC) for predicting the postoperative recurrence of non-functioning pituitary adenomas. The DeLong test was used to compare the predictive performance of different models. Utilizing decision curve analysis(DCA), the clinical utility of different models was evaluated by quantifying the net benefit at various probability thresholds. Finally, the different predictive models were validated in the validation set and an external testing set^18^.

### Statistical methods

Statistical analysis of clinical and MRI features was conducted using SPSS (version 26.0). For the quantitative data of patients’ age, tumor T1, T2, and enhanced signal intensity ratios, maximum diameter of the tumor, and tumor volume, a normality test was first performed. For factors that met the normal distribution, an independent samples t-test was used for analysis, while the Mann-Whitney U test was used for those that did not meet the normal distribution. The chi-square test was used for categorical variables. Single-factor and multi-factor logistic regression analyses were used to screen for clinical, MRI, and pathological features related to the postoperative recurrence of pituitary tumors. The stepwise backward regression method was applied with AIC as the criterion, and finally, a logistic regression model was constructed.

For radiomics analysis, R software (version 4.2.2) was used. The “glmnet” package (version 4.1-8) was employed for Lasso regression analysis to select features. Based on the calculated Cli-score, Rad-score, and Combined score, ROC curves were plotted, and the AUC value was calculated. The “rms” package (version 6.9-0) in R software was used to draw the nomogram and calibration curve, and the “rmda” package (version 1.6) was used to draw the DCA curve to evaluate the clinical application value of the model.

## Results

### Clinical and conventional imaging features

The clinical, pathological, and conventional imaging features of the three groups are shown in Table 1. There was no statistical difference in clinical features (gender, age, symptoms, surgical resection range), conventional imaging features (invasion site, morphology, cystic change, hemorrhage, signal uniformity, Knosp grading, volume, diameter, T1 signal intensity ratio, T1 enhancement signal intensity ratio), and pathological classification (between ACTH, PRL, TSH, LH, GH) between the training set, validation set, and external test set (P values ranged from 0.067 to 0.705). There were statistical differences between tumor texture, T2 signal intensity ratio, FSH positivity, and Ki-67 index (P values ranged from 0.000 to 0.001). There was no statistical difference in tumor recurrence rate between the three groups (*P* = 0.261).

Tables 2, 3 and 4 show the relationship between NFMA recurrence and the clinical-imaging-pathological features of patients in the training set, validation set, and external test set, respectively. Normality tests were performed on quantitative data such as patient age, tumor volume, diameter, T1 signal intensity ratio, T2 signal intensity ratio, and T1 enhancement signal intensity ratio. The results showed that age was normally distributed in the recurrence group (*P* = 0.825), while the others were not normally distributed. Therefore, the rank-sum test was used for statistical analysis of the quantitative data. In the training set, there was a statistical difference in tumor volume (10.1 ± 10.9cm3 vs. 13.0 ± 9.0cm3, *P*<0.001) and diameter (26.6 ± 11.1 mm vs. 28.9 ± 9.4 mm, *P* = 0.012) between the non-recurrence group and the recurrence group. The recurrence group was larger than the non-recurrence group. In the validation set, the volume and diameter of the recurrence group (12.5 ± 10cm3, 29.2 ± 8.6 mm) were larger than those of the non-recurrence group (9.8 ± 7.8cm3, 26 ± 9.3 mm), but there was no statistical significance (P values were 0.075 and 0.109, respectively). In the external test set, the volume and diameter of the recurrence group (16.7 ± 12.5cm3, 28.5 ± 8.2 mm) were larger than those of the non-recurrence group (9.8 ± 6.3cm3, 24.8 ± 6.7 mm), and the differences were statistically significant (P values were 0.003 and 0.029, respectively). There was no statistical difference in age, T1 signal intensity ratio, T2 signal intensity ratio, and T1 enhancement signal intensity ratio between the recurrence group and the non-recurrence group in the training set, validation set, and external test set.

**Table 1:** Clinical and MR features in the training set, validation set, and external test set.

Variable		Training Set (*n* = 204)	Validation Set (*n* = 88)	External Test Set (*n* = 123)	*P*-value
Gender					0.538
	Male	85 (41.7%)	39 (44.3%)	59 (48%)	
	Female	119 (58.3%)	49 (55.7%)	64 (52%)	
Age (years)		54.4 ± 12.4	51 ± 14.1	45.3 ± 12.8	0.069
Symptom					0.071
	None	6 (2.9%)	18 (20.5%)	18 (14.6%)	
	Dizziness	7 (3.4%)	6 (6.8%)	12 (9.8%)	
	Blurred vision	149 (73%)	61 (69.3%)	78 (63.4%)	
	Headache	42 (20.6%)	3 (3.4%)	15 (12.2%)	
Consistency					0.000
	Softness	137 (67.2%)	68 (77.3%)	78 (63.4%)	
	Tough	67 (32.8%)	20 (22.7%)	45 (36.6%)	
Invasive site					0.094
	None	24 (11.8%)	11 (12.5%)	9 (7.3%)	
	Suprasellar	88 (43.1%)	48 (54.5%)	56 (45.5%)	
	Parasellar	12 (5.9%)	8 (9.1%)	5 (4.1%)	
	Sphenoid sinus	15 (7.4%)	6 (6.8%)	6 (4.9%)	
	Supra + Parasellar	49 (24%)	12 (13.6%)	22 (17.9%)	
	Supra + Parasellar + Sphenoid	16 (7.8%)	3 (3.4%)	25 (20.3%)	
Morphology					0.67
	Oval	23 (11.3%)	24 (27.3%)	33 (26.8%)	
	Dumbbell	43 (21.1%)	23 (26.1%)	42 (34.1%)	
	Irregular	75 (36.8%)	27 (30.7%)	27 (22%)	
	Lobulated	23 (11.3%)	1 (1.1%)	11 (8.9%)	
Cystic change					0.074
	None	105 (51.5%)	43 (48.9%)	55 (44.7%)	
	Small cystic	54 (26.5%)	17 (19.3%)	22 (17.9%)	
	Moderate cystic	13 (6.4%)	6 (6.8%)	23 (18.7%)	
	Large cystic	25 (12.3%)	15 (17%)	19 (15.4%)	
	Complete cystic	7 (3.4%)	7 (8%)	4 (3.3%)	
Hemorrhage					0.405
	None	147 (72.1%)	70 (79.5%)	91 (74%)	
	Present	57 (27.9%)	18 (20.5%)	32 (26%)	
Signal					0.322
	Homogeneous	77 (37.7%)	39 (44.3%)	42 (34.1%)	
	Heterogeneous	127 (62.3%)	49 (55.7%)	81 (65.9%)	
Knosp grading					0.071
	0	18 (8.8%)	4 (4.5%)	2 (1.6%)	
	1	43 (21.1%)	18 (20.5%)	21 (17.1%)	
	2	57 (27.9%)	42 (47.7%)	57 (46.3%)	
	3	54 (26.5%)	19 (21.6%)	29 (23.6%)	
	4	32 (15.7%)	5 (5.7%)	14 (11.4%)	
Resection range					0.163
	Gross total	116 (56.9%)	66 (75%)	101 (82.1%)	
	Subtotal	66 (32.4%)	19 (21.6%)	20 (16.3%)	
	Partial resection	22 (10.8%)	3 (3.4%)	2 (1.6%)	
ACTH					0.682
	-	176 (86.3%)	73 (83%)	107 (87%)	
	+	28 (13.7%)	15 (17%)	16 (13%)	
FSH					0.000
	-	121 (59.3%)	50 (56.8%)	102 (82.9%)	
	+	83 (40.7%)	38 (43.2%)	21 (17.1%)	
LH					0.079
	-	194 (95.1%)	79 (89.8%)	119 (96.7%)	
	+	10 (4.9%)	9 (10.2%)	4 (3.3%)	
TSH					0.351
	-	186 (91.2%)	76 (86.4%)	113 (91.9%)	
	+	18 (8.8%)	12 (13.6%)	10 (8.1%)	
GH					0.175
	-	178 (87.3%)	76 (86.4%)	80 (65%)	
	+	26 (12.7%)	12 (13.6%)	43 (35%)	
PRL					0.569
	-	152 (74.5%)	64 (72.7%)	85 (69.1%)	
	+	52 (25.5%)	24 (27.3%)	38 (30.9%)	
Ki−67					0.001
	Ki−67 < 3	137 (67.2%)	57 (64.8%)	58 (47.2%)	
	Ki−67 ≥ 3	67 (32.8%)	31 (35.2%)	65 (52.8%)	
Hormone type					0.441
	Non-hormonal	56 (27.5%)	18 (20.5%)	33 (26.8%)	
	Single hormone	105 (51.5%)	43 (48.9%)	60 (48.8%)	
	Multiple hormones	43 (21.1%)	27 (30.7%)	30 (24.4%)	
Volume (cm3)		11.3 ± 10.3	10.9 ± 8.9	12 ± 9.3	0.705
Diameter (mm)		27.5 ± 10.5	27.3 ± 9.1	26.1 ± 7.4	0.155
T1 signal ratio		1.2 ± 0.14	1 ± 0.1	1 ± 0.2	0.515
T2 signal ratio		1.6 ± 0.3	1.6 ± 0.4	1.8 ± 0.4	0.000
T1 enhanced signal ratio		1.7 ± 0.4	1.7 ± 0.4	1.6 ± 0.5	0.069

Note: Count data are presented as frequency (percentage); measurement data are presented as mean ± standard deviation.

**Table 2:** Relationship between Basic Clinical and MR features in the training set and postoperative recurrence.

Variable		No Recurrence (*n* = 120)	Recurrence (*n* = 84)	*P*-value
Gender				0.149
	Male	55 (45.8%)	30 (35.7%)	
	Female	65 (54.2%)	54 (64.3%)	
Age (years)		55.4 ± 12.4	53 ± 12.5	0.185
Symptom				0.287
	None	4 (3.3%)	2 (2.4%)	
	Dizziness	4 (3.3%)	3 (3.6%)	
	Blurred vision	82 (68.3%)	67 (79.8%)	
	Headache	30 (25%)	12 (14.3%)	
Consistency				0.277
	Softness	77 (64.2%)	60 (71.4%)	
	Tough	43 (35.8%)	24 (28.6%)	
Invasion site				0.001
	None	19 (15.8%)	5 (6%)	
	Suprasellar	53 (44.2%)	35 (41.7%)	
	Parasellar	6 (5%)	6 (7.1%)	
	Sellar	14 (11.7%)	1 (1.2%)	
	Suprasellar + Parasellar	24 (20%)	25 (29.8%)	
	Suprasellar + Parasellar + Sellar	4 (3.3%)	12 (14.3%)	
Shape				0.000
	Round	33 (27.5%)	7 (8.3%)	
	Oval	16 (13.3%)	7 (8.3%)	
	Dumbbell	25 (20.8%)	18 (21.4%)	
	Irregular	40 (33.3%)	35 (41.7%)	
	Lobulated	6 (5%)	17 (20.2%)	
Cystic change				0.434
	None	65 (54.2%)	40 (47.6%)	
	Small cystic change	28 (23.3%)	26 (31%)	
	Moderate cystic change	7 (5.8%)	6 (7.1%)	
	Large cystic change	14 (11.7%)	11 (13.1%)	
	Complete cystic change	6 (5%)	1 (1.2%)	
Hemorrhage				0.271
	None	83 (69.2%)	64 (76.2%)	
	Present	37 (30.8%)	20 (23.8%)	
Signal				0.501
	Homogeneous	43 (35.8%)	34 (40.5%)	
	Heterogeneous	77 (64.2%)	50 (59.5%)	
Knosp grade				0.002
	0	15 (12.5%)	3 (3.6%)	
	1	33 (27.5%)	10 (11.9%)	
	2	32 (26.7%)	25 (29.8%)	
	3	28 (23.3%)	26 (31%)	
	4	12 (10%)	20 (23.8%)	
Resection range				0.000
	Total resection	90 (75%)	26 (31%)	
	Subtotal resection	29 (24.2%)	37 (44%)	
	Large partial resection	1 (0.8%)	21 (25%)	
ACTH				0.307
	-	106 (88.3%)	70 (83.3%)	
	+	14 (11.7%)	14 (16.7%)	
FSH				0.358
	-	68 (56.7%)	53 (63.1%)	
	+	52 (43.3%)	31 (36.9%)	
LH				0.561
	-	115 (95.8%)	79 (94%)	
	+	5 (4.2%)	5 (6%)	
TSH				0.965
	-	110 (91.7%)	76 (90.5%)	
	+	10 (8.3%)	8 (9.5%)	
GH				0.004
	-	98 (81.7%)	80 (95.2%)	
	+	22 (18.3%)	4 (4.8%)	
PRL				0.036
	-	83 (69.2%)	69 (82.1%)	
	+	37 (30.8%)	15 (17.9%)	
Ki−67				0.181
	Ki−67 < 3	85 (70.8%)	52 (61.9%)	
	Ki−67 ≥ 3	35 (29.2%)	32 (38.1%)	
Hormone type				0.259
	Non-hormone type	28 (23.3%)	28 (33.3%)	
	Single hormone type	64 (53.3%)	41 (48.8%)	
	Multiple hormone type	28 (23.3%)	15 (17.9%)	
Volume (cm3)		10.1 ± 10.9	13 ± 9	0.000
Diameter (mm)		26.6 ± 11.1	28.9 ± 9.4	0.012
T1 signal ratio		1.3 ± 0.1	1 ± 0.1	0.761
T2 signal ratio		1.5 ± 0.3	1.6 ± 0.4	0.061
T1 enhanced signal ratio		1.7 ± 0.4	1.7 ± 0.4	0.146

Note: Count data are presented as frequency (percentage); measurement data are presented as mean ± standard deviation.

**Table 3:** Relationship between Basic Clinical and MR features in the Validation Set and Postoperative Recurrence.

Variable		No Recurrence (*n* = 52)	Recurrence (*n* = 36)	*P*-value
Gender				0.648
	Male	22 (42.3%)	17 (47.2%)	
	Female	30 (57.7%)	19 (52.8%)	
Age (years)		50.8 ± 14.5	51.3 ± 13.6	0.877
Symptom				0.000
	None	18 (34.6%)	0 (0%)	
	Dizziness	1 (1.9%)	5 (13.9%)	
	Blurred vision	30 (57.7%)	31 (86.1%)	
	Headache	3 (5.8%)	0 (0%)	
Consistency				0.013
	Softness	45 (86.5%)	23 (63.9%)	
	Tough	7 (13.5%)	13 (36.1%)	
Invasion site				0.194
	None	9 (17.3%)	2 (5.6%)	
	Suprasellar	27 (51.9%)	21 (58.3%)	
	Parasellar	5 (9.6%)	3 (8.3%)	
	Sellar	5 (9.6%)	1 (2.8%)	
	Suprasellar + Parasellar	4 (7.7%)	8 (22.2%)	
	Suprasellar + Parasellar + Sellar	2 (3.8%)	1 (2.8%)	
Shape				0.000
	Round	13 (25%)	0 (0%)	
	Oval	23 (44.2%)	1 (2.8%)	
	Dumbbell	12 (23.1%)	11 (30.6%)	
	Irregular	4 (7.7%)	23 (63.9%)	
	Lobulated	0 (0%)	1 (2.8%)	
Cystic change				0.336
	None	29 (55.8%)	14 (38.9%)	
	Small cystic change	8 (15.4%)	9 (25%)	
	Moderate cystic change	2 (3.8%)	4 (11.1%)	
	Large cystic change	8 (15.4%)	7 (19.4%)	
	Complete cystic change	5 (9.6%)	2 (5.6%)	
Hemorrhage				0.845
	None	41 (78.8%)	29 (80.6%)	
	Present	11 (21.2%)	7 (19.4%)	
Signal				0.394
	Homogeneous	25 (48.1%)	14 (38.9%)	
	Heterogeneous	27 (51.9%)	22 (61.1%)	
Knosp grade				0.000
	0	4 (7.7%)	0 (0%)	
	1	16 (30.8%)	2 (5.6%)	
	2	28 (53.8%)	14 (38.9%)	
	3	4 (7.7%)	15 (41.7%)	
	4	0 (0%)	5 (13.9%)	
Resection range				0.073
	Total resection	42 (80.8%)	24 (66.7%)	
	Subtotal resection	10 (19.2%)	9 (25%)	
	Large partial resection	0 (0%)	3 (8.3%)	
ACTH				0.071
	-	40 (76.9%)	33 (91.7%)	
	+	12 (23.1%)	3 (8.3%)	
FSH				0.131
	-	33 (63.5%)	17 (47.2%)	
	+	19 (36.5%)	19 (52.8%)	
LH				0.229
	-	45 (86.5%)	34 (94.4%)	
	+	7 (13.5%)	2 (5.6%)	
TSH				0.014
	-	41 (78.8%)	35 (97.2%)	
	+	11 (21.2%)	1 (2.8%)	
GH				0.228
	-	43 (82.7%)	33 (91.7%)	
	+	9 (17.3%)	3 (8.3%)	
PRL				0.17
	-	35 (67.3%)	29 (80.6%)	
	+	17 (32.7%)	7 (19.4%)	
Ki−67				0.885
	Ki−67 < 3	34 (65.4%)	23 (63.9%)	
	Ki−67 ≥ 3	18 (34.6%)	13 (36.1%)	
Hormone type				0.048
	Non-hormone type	8 (15.4%)	10 (27.8%)	
	Single hormone type	23 (44.2%)	20 (55.6%)	
	Multiple hormone type	21 (40.4%)	6 (16.7%)	
Volume (cm3)		9.8 ± 7.8	12.5 ± 10	0.075
Diameter (mm)		26 ± 9.3	29.2 ± 8.6	0.109
T1 signal ratio		1 ± 0.1	1 ± 0.1	0.06
T2 signal ratio		1.5 ± 0.4	1.7 ± 0.3	0.005
T1 enhanced signal ratio		1.7 ± 0.4	1.7 ± 0.4	0.665

Note: Count data are presented as frequency (percentage); measurement data are presented as mean ± standard deviation.

**Table 4:** Relationship between Basic Clinical and MR features in the External Test Set and Postoperative Recurrence.

Variable		No Recurrence (*n* = 52)	Recurrence (*n* = 36)	*P*-value
Gender				0.648
	Male	22(42.3%)	17(47.2%)	
	Female	30(57.7%)	19(52.8%)	
Age (years)		50.8 ± 14.5	51.3 ± 13.6	0.877
Symptom				0.000
	None	18(34.6%)	0(0%)	
	Dizziness	1(1.9%)	5(13.9%)	
	Blurred vision	30(57.7%)	31(86.1%)	
	Headache	3(5.8%)	0(0%)	
Consistency				0.013
	Softness	45(86.5%)	23(63.9%)	
	Tough	7(13.5%)	13(36.1%)	
Invasion site				0.194
	None	9(17.3%)	2(5.6%)	
	Suprasellar	27(51.9%)	21(58.3%)	
	Parasellar	5(9.6%)	3(8.3%)	
	Sellar	5(9.6%)	1(2.8%)	
	Suprasellar + Parasellar	4(7.7%)	8(22.2%)	
	Suprasellar + Parasellar + Sellar	2(3.8%)	1(2.8%)	
Shape				0.000
	Round	13(25%)	0(0%)	
	Oval	23(44.2%)	1(2.8%)	
	Dumbbell	12(23.1%)	11(30.6%)	
	Irregular	4(7.7%)	23(63.9%)	
	Lobulated	0(0%)	1(2.8%)	
Cystic change				0.336
	None	29(55.8%)	14(38.9%)	
	Small cystic change	8(15.4%)	9(25%)	
	Moderate cystic change	2(3.8%)	4(11.1%)	
	Large cystic change	8(15.4%)	7(19.4%)	
	Complete cystic change	5(9.6%)	2(5.6%)	
Hemorrhage				0.845
	None	41(78.8%)	29(80.6%)	
	Present	11(21.2%)	7(19.4%)	
Signal				0.394
	Homogeneous	25(48.1%)	14(38.9%)	
	Heterogeneous	27(51.9%)	22(61.1%)	
Knosp grade				0.000
	0	4(7.7%)	0(0%)	
	1	16(30.8%)	2(5.6%)	
	2	28(53.8%)	14(38.9%)	
	3	4(7.7%)	15(41.7%)	
	4	0(0%)	5(13.9%)	
Resection range				0.073
	Total resection	42(80.8%)	24(66.7%)	
	Subtotal resection	10(19.2%)	9(25%)	
	Large partial resection	0(0%)	3(8.3%)	
ACTH				0.071
	-	40(76.9%)	33(91.7%)	
	+	12(23.1%)	3(8.3%)	
FSH				0.131
	-	33(63.5%)	17(47.2%)	
	+	19(36.5%)	19(52.8%)	
LH				0.229
	-	45(86.5%)	34(94.4%)	
	+	7(13.5%)	2(5.6%)	
TSH				0.014
	-	41(78.8%)	35(97.2%)	
	+	11(21.2%)	1(2.8%)	
GH				0.228
	-	43(82.7%)	33(91.7%)	
	+	9(17.3%)	3(8.3%)	
PRL				0.17
	-	35(67.3%)	29(80.6%)	
	+	17(32.7%)	7(19.4%)	
Ki−67				0.885
	Ki−67 < 3	34(65.4%)	23(63.9%)	
	Ki−67 ≥ 3	18(34.6%)	13(36.1%)	
Hormone type				0.048
	Non-hormonal	8(15.4%)	10(27.8%)	
	Single hormone type	23(44.2%)	20(55.6%)	
	Multiple hormone type	21(40.4%)	6(16.7%)	
Volume (cm3)		9.8 ± 7.8	12.5 ± 10	0.075
Diameter (mm)		26 ± 9.3	29.2 ± 8.6	0.109
T1 signal ratio		1 ± 0.1	1 ± 0.1	0.06
T2 signal ratio		1.5 ± 0.4	1.7 ± 0.3	0.005
T1 enhanced signal ratio		1.7 ± 0.4	1.7 ± 0.4	0.665

Note: Count data are presented as frequency (percentage); measurement data are presented as mean ± standard deviation.

Qualitative data included the patient’s gender, symptoms, degree of tumor resection, tumor texture, invasive site of the tumor, morphology, presence of intratumoral hemorrhage, signal uniformity, cystic changes, Knosp grading, immunohistochemical hormone type, and Ki-67 proliferation index. In the training set, there were statistically significant differences between the non-recurrence group and the recurrence group in terms of tumor invasion site (*P* = 0.001), tumor morphology (*P*<0.001), Knosp grading (*P* = 0.002), tumor resection range (*P*<0.001), immunohistochemical GH positivity (*P* = 0.004), and immunohistochemical PRL positivity (*P* = 0.036). Those with a larger range of invasion, more irregular morphology, higher Knosp grading, incomplete resection, and negative GH and PRL immunohistochemistry were more likely to experience recurrence. In the validation set, there were still statistical differences between the non-recurrence group and the recurrence group in terms of tumor morphology (*P*<0.001) and Knosp grading (<0.001). Although those with a larger range of invasion, incomplete resection, and negative GH and PRL immunohistochemistry were still more likely to experience recurrence in the validation set, there was no statistical significance between the recurrence group and the non-recurrence group in terms of tumor invasion site (*P* = 0.194), tumor resection range (*P*<0.073), immunohistochemical GH positivity (*P* = 0.228), and immunohistochemical PRL positivity (*P* = 0.17). In the external test set, there were still statistical differences between the non-recurrence group and the recurrence group in terms of tumor morphology (*P*<0.001), Knosp grading (<0.001), tumor resection range (*P* = 0.002), and immunohistochemical GH positivity (*P* = 0.016). Although those with a larger range of invasion and negative PRL immunohistochemistry were still more likely to experience recurrence in the external test set, there was no statistical significance between the recurrence group and the non-recurrence group in terms of tumor invasion site (*P* = 0.113) and immunohistochemical PRL positivity (*P* = 0.162).

### Construction and validation of clinical model

Using the training set data, a clinical model predicting postoperative recurrence of non-functional pituitary adenomas was established. Firstly, single-factor Logistics regression analysis of clinical-imaging-pathological features of the recurrence and non-recurrence groups in the training set was performed (as shown in Table 5), and potential risk factors were screened out. Then, multi-factor Logistics regression analysis was conducted, using stepwise backward elimination method and AIC as the stopping rule to adjust the independent variables in the model, thereby selecting the optimal feature subset. The following factors were identified as risk factors for predicting tumor recurrence after surgery: dumbbell-shaped tumor morphology (OR = 4.981, *P* = 0.031), irregular morphology (OR = 4.816, *P* = 0.039), lobulated shape (OR = 10.446, *P* = 0.022), subtotal resection (OR = 3.522, *P* = 0.002), major resection (OR = 43.685, *P* = 0.001), PRL positivity (OR = 0.226, *P* = 0.007), Ki-67 ≧ 3 (OR = 3.14, *P* = 0.042), T2 signal ratio (OR = 3.17, *P* = 0.046). Using the above features to establish a clinical model, the clinical score (Cli-score) was calculated. The calculation formula is as follows:

**Table 5:** Univariate and Multivariate Logistic Regression Analysis of Clinical, Imaging, and pathological features in the Training Set.

Feature	Univariate		Multivariate	
	OR( 95%CI)	*P*-value	OR( 95%CI)	*P*-value
Male	1.523(0.859–2.701)	0.15		
Age	0.985(0.963–1.007)	0.185		
Symptoms		0.294		
Headache	1.5(0.156–14.42)	0.725		
Dizziness	1.634(0.29–9.197)	0.577		
Blurred vision	0.8(0.129–4.96)	0.811		
Consistency	0.716(0.392–1.309)	0.278		
Volume	1.028(0.999–1.059)	0.062		
Invasion Site		0.004		0.206
Suprasellar	2.509(0.858–7.343)	0.093	0.822(0.183–3.696)	0.798
Parasellar	3.8(0.848–17.036)	0.081	1.653(0.202–13.539)	0.64
Cavernous sinus	0.271(0.028–2.589)	0.257	0.046(0.003–0.851)	0.059
Supra + Parasellar	3.958(1.275–12.293)	0.017	0.565(0.089–3.573)	0.544
Supra + Parasellar + Cavernous	11.4(2.543–51.108)	0.001	1.221(0.106–14.073)	0.873
Morphology		0.001		0.138
Ellipsoid	2.062(0.618–6.888)	0.239	2.675(0.518–13.806)	0.24
Dumbbell	3.394(1.229–9.375)	0.018	4.981(1.157–21.437)	0.031
Irregular	4.125(1.622–10.489)	0.003	4.816(1.081–21.455)	0.039
Lobulated	13.357(3.875–46.042)	<0.001	10.446(1.41-77.399)	0.022
Cystic		0.485		
Small cyst	1.509(0.777–2.929)	0.224		
Moderate cyst	1.393(0.437–4.44)	0.575		
Large cyst	1.277(0.528–3.086)	0.587		
Complete cyst	0.271(0.031–2.333)	0.234		
Hemorrhage	0.701(0.372–1.322)	0.272		
Heterogeneous signal	0.821(0.463–1.457)	0.501		
Diameter	1.021(0.994–1.049)	0.129		
Knosp grading	1.679(1.297–2.175)	<0.001	0.922(0.586–1.451)	0.725
Resection range		<0.001		<0.001
Subtotal resection	4.416(2.298–8.487)	<0.001	3.522(1.581–7.844)	0.002
Major resection	72.692(9.33-566.364)	<0.001	43.685(4.896-389.779)	0.001
ACTH(+)	1.514(0.68–3.37)	0.309		
FSH(+)	0.765(0.432–1.355)	0.358		
LH(+)	1.456(0.408–5.195)	0.563		
TSH(+)	1.158(0.437–3.068)	0.768		
GH(+)	0.223(0.074–0.673)	0.008	0.301(0.07–1.285)	0.105
PRL(+)	0.488(0.247–0.962)	0.038	0.226(0.077–0.663)	0.007
Ki67 ≥ 3	2.767(2.228–2.898)	0.035	3.14(1.962–5.761)	0.042
Hormone type		0.262		
Single hormone type	0.641(0.333–1.232)	0.182		
Multiple hormone type	0.536(0.237–1.213)	0.134		
T1 signal ratio	0.346(0.036–3.316)	0.357		
T2 signal ratio	2.905(1.246–6.773)	0.014	3.17(1.019–9.864)	0.046
T1 enhanced signal ratio	0.773(0.385–1.555)	0.471		

Cli-score = -3.4258 + (dumbbell shape × 0.9588) + (irregular shape × 0.7815) + (lobulated shape × 1.3755) + (subtotal resection × 1.3370) + (major resection × 4.0508) + (PRL × -1.0205) + (Ki-67 × 0.6355) + (T2 signal ratio × 1.0318).

**Fig. 2:**
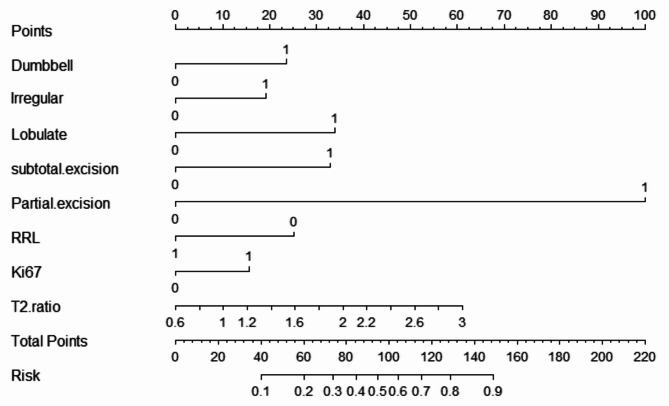
Nomogram plotted based on the clinical characteristics of the patient to predict the postoperative recurrence of non-functioning pituitary adenoma. The corresponding scale represents the value of each variable. The total score can be obtained by adding the scores of the two variables. The predictive probability corresponding to the scale below can be found.

**Fig. 3:**
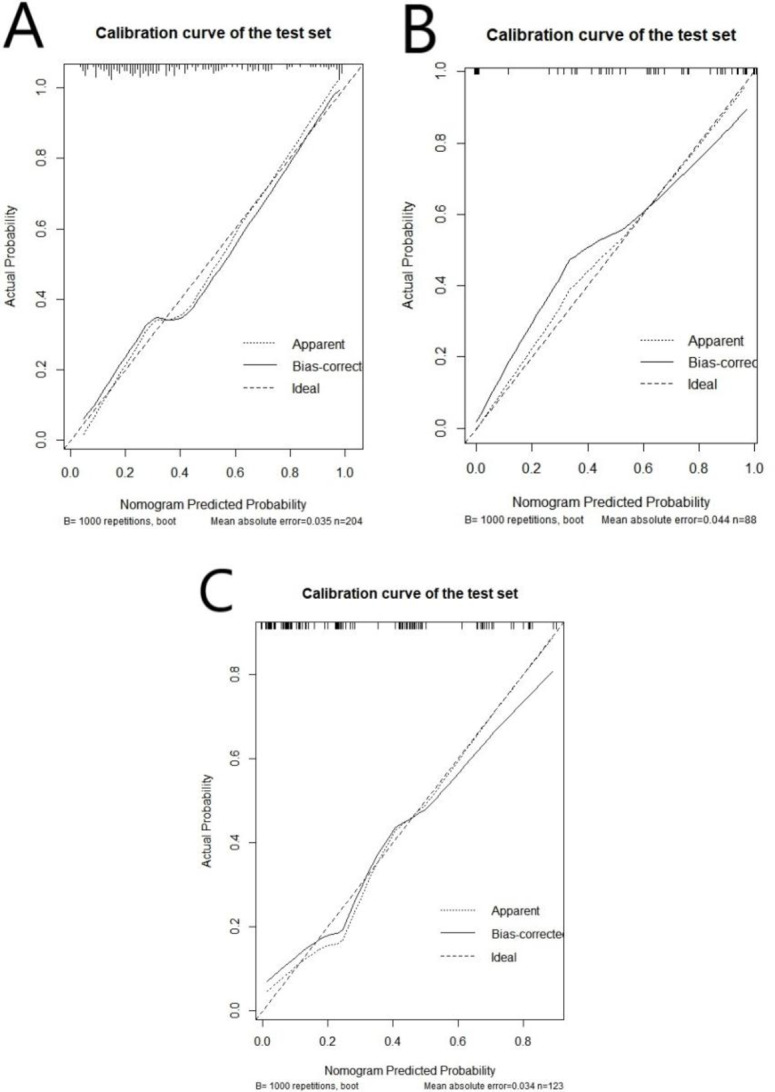
Calibration curve of the clinical prediction model in the training set (A), validation set (B), and external test set (C).

The nomogram of the clinical prediction model was plotted (as shown in Fig. 2). Through the nomogram, the scores corresponding to different clinical features can be intuitively displayed. The total score is obtained by adding the scores of each feature, and the bottommost row represents the probability of postoperative recurrence of non-functional pituitary adenomas. The calibration curve (Fig. 3) was plotted to evaluate the predictive effect of the nomogram. The vertical axis represents the true probability, the horizontal axis represents the predicted probability, the solid line represents the actual predicted value, and the dashed line represents the ideal predicted value. The closer the two lines are, the better the prediction effect. The results showed that the predictive curves of the clinical model in the training set, validation set, and external test set were well-fitted with the standard curve, indicating a good predictive effect of the model.

The diagnostic performance of the clinical model is shown in Table 6. The AUC of the Cli-score model in the training set was 0.834 (95%CI: 0.775–0.882), with SEN, SPE, ACC of 78.57%, 70.83%, 75.49%, respectively (as shown in Table 5); in the validation set, the AUC was 0.756 (95%CI: 0.653–0.841), with SEN, SPE, ACC of 83.33%, 57.69%, 70.45%; and in the external test set, the AUC was 0.757 (95%CI: 0.671–0.830), with SEN, SPE, ACC of 82.5%, 59.04%, 71.54%.

**Table 6:** Diagnostic performance of Cli-score, Rad-score, and combined model.

Model	Cohort	AUC (95% CI)	SEN	SPE	ACC
Cli-score	Training set	0.834 (0.775–0.882)	78.57%	70.83%	75.49%
	Validation set	0.756 (0.653–0.841)	83.33%	57.69%	70.45%
	External test set	0.757 (0.671–0.830)	82.5%	59.04%	71.54%
Rad-score	Training set	0.88 (0.827–0.921)	83.33%	77.5%	81.37%
	Validation set	0.841 (0.748–0.911)	72.22%	86.54%	69.32%
	External test set	0.835 (0.757–0.896)	80%	79.52%	63.41%
Combined model	Training set	0.908 (0.860–0.944)	85.71%	81.67%	83.33%
	Validation set	0.888 (0.803–0.945)	77.78%	86.54%	79.55%
	External test set	0.863 (0.790–0.919)	80%	81.93%	68.30%

### Construction and validation of radiomics model

**Fig. 4:**
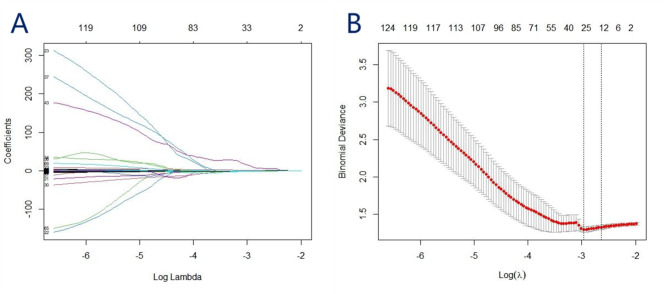
A is the convergence diagram of feature coefficients, with the vertical axis representing the coefficients of different radiomic features and the horizontal axis representing Log(λ). The feature coefficients change with the variation of λ. B is the curve diagram of 10-fold cross-validation. The optimal λ value is selected using 10-fold cross-validation to minimize the bias of the model, with the vertical dashed line indicating the Log(λ) corresponding to the optimal λ value.

From T1WI, T2WI, and T1-enhanced MR images of patients with non-functional pituitary adenomas, a total of 3390 radiomic features were extracted. Through consistency testing, 356 radiomic features with ICCs < 0.75 were removed, leaving 3034 radiomic features. The remaining radiomic features were included in the subsequent analysis. Univariate selection of features was conducted using variance analysis, selecting 1535 features with *P* < 0.05. Subsequently, based on LASSO regression combined with the AIC method, 14 features were selected from the three sequences, with 6, 5, and 3 features related to the recurrence of non-functional pituitary adenomas after surgery selected from the T1-enhanced, T1WI, and T2WI sequences, respectively (Table 7). Figure 4 illustrates the process of LASSO regression selecting radiomic features.

**Table 7:** Radiomic features selected from different MR sequences.

MR Sequence	Number	Feature
T1 enhanced	6	original_firstorder_Skewness
		original_glcm_MCC
		log-sigma-1-0-mm-3D_glcm_Correlation
		wavelet-LHL_gldm_LargeDependenceHighGrayLevelEmphasis
		wavelet-HLH_glcm_ClusterProminence
		wavelet-HLH_glszm_SmallAreaLowGrayLevelEmphasis
T1	5	original_shape_Elongation
		wavelet-LHH_firstorder_Skewness
		wavelet-HLL_glcm_JointEnergy
		wavelet-HLL_glrlm_RunVariance
		wavelet-HHL_firstorder_Kurtosis
T2	3	wavelet-LLH_glcm_MCC
		wavelet-HLL_firstorder_Skewness
		wavelet-HHH_firstorder_Median

Using logistic regression with the final selected 14 features, a prediction model was established to compute the radiomic score (Rad-score):

Rad-score = 3.1

+ 1.273×T1 + C_original_firstorder_Skewness

-7.706×T1 + C_original_glcm_MCC

-12.11×T1 + C_log-sigma-1-0-mm-3D_glcm_Correlation

+ 0.00002813×T1 + C_wavelet-LHL_gldm_LargeDependenceHighGrayLevelEmphasis

-0.004478×T1 + C_wavelet-HLH_glcm_ClusterProminence

+ 9.249×T1 + C_wavelet-HLH_glszm_SmallAreaLowGrayLevelEmphasis

-4.812×T1_original_shape_Elongation

+ 1.338×T1_wavelet-LHH_firstorder_Skewness

+ 92.85×T1_wavelet-HLL_glcm_JointEnergy

-3.93×T1_wavelet-HLL_glrlm_RunVariance

+ 0.09704×T1_wavelet-HHL_firstorder_Kurtosis

+ 15.14×T2_wavelet-LLH_glcm_MCC

+ 1.161×T2_wavelet-HLL_firstorder_Skewness

+ 5.79×T2_wavelet-HHH_firstorder_Median

According to the above calculation formula, the Rad-score for each patient with non-functional pituitary adenomas was computed. The AUC of this model in the training set was 0.88 (95% CI: 0.827–0.921), with SEN, SPE, and ACC of 83.33%, 77.5%, and 81.37%, respectively (Table 5); in the validation set, the AUC of the Rad-score model was 0.841 (95% CI: 0.748–0.911), with SEN, SPE, and ACC of 72.22%, 86.54%, and 69.32%; and in the external test set, the AUC was 0.835 (95% CI: 0.757–0.896), with SEN, SPE, and ACC of 80%, 79.52%, and 63.41%.

### Construction and validation of the combined prediction model

**Fig. 5:**
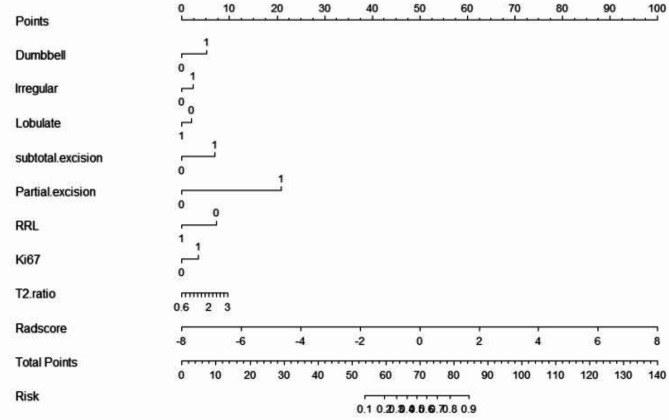
combined model nomogram plotted based on the clinical characteristics of the patient and Rad-score to predict the postoperative recurrence of non-functioning pituitary adenoma.

**Fig. 6:**
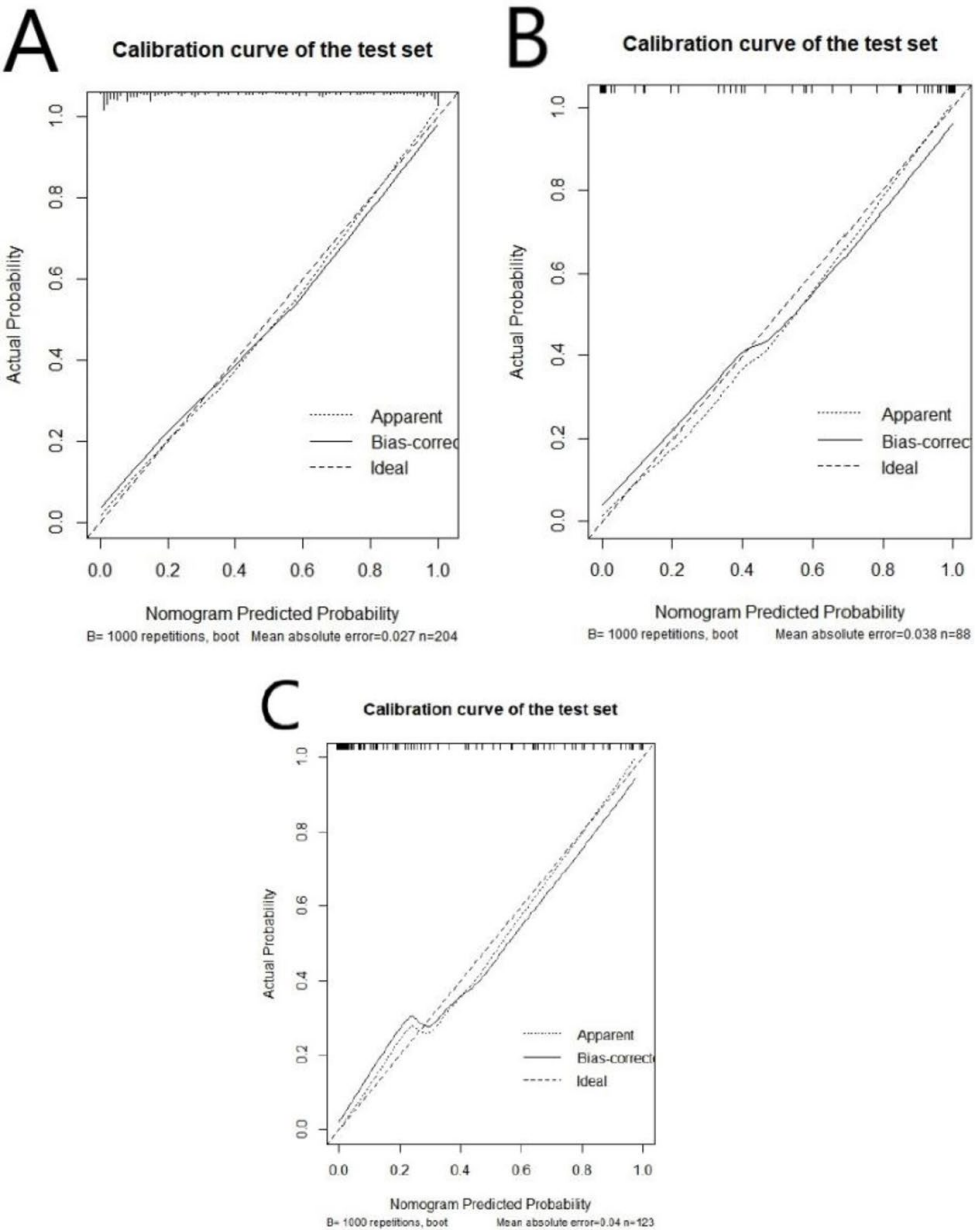
Calibration curve of the combined prediction model in the training set (A), validation set (B), and external test set (C).

We combined the clinical-imaging-pathological features of patients with non-functional pituitary adenomas and the Rad-score to establish a combined prediction model for predicting the postoperative recurrence of non-functional pituitary adenomas using logistic regression and plotted a nomogram (Fig. 5). The AUC of this model in the training set was 0.908 (95% CI: 0.860–0.944), with SEN, SPE, and ACC of 85.71%, 81.67%, and 83.33%, respectively (Table 5); in the validation set, the AUC of the combined model was 0.888 (95% CI: 0.803–0.945), with SEN, SPE, and ACC of 77.78%, 86.54%, and 79.55%; and in the external test set, the AUC was 0.863 (95% CI: 0.790–0.919), with SEN, SPE, and ACC of 80%, 81.93%, and 68.30%. A calibration curve (Fig. 6) was plotted to evaluate the predictive performance of the nomogram, and the results showed a good fit between the prediction curve of the combined prediction model and the standard curve in the training set, validation set, and external test set, indicating a good predictive performance of the model.

### Comparison of prediction performance of different models

**Fig. 7:**
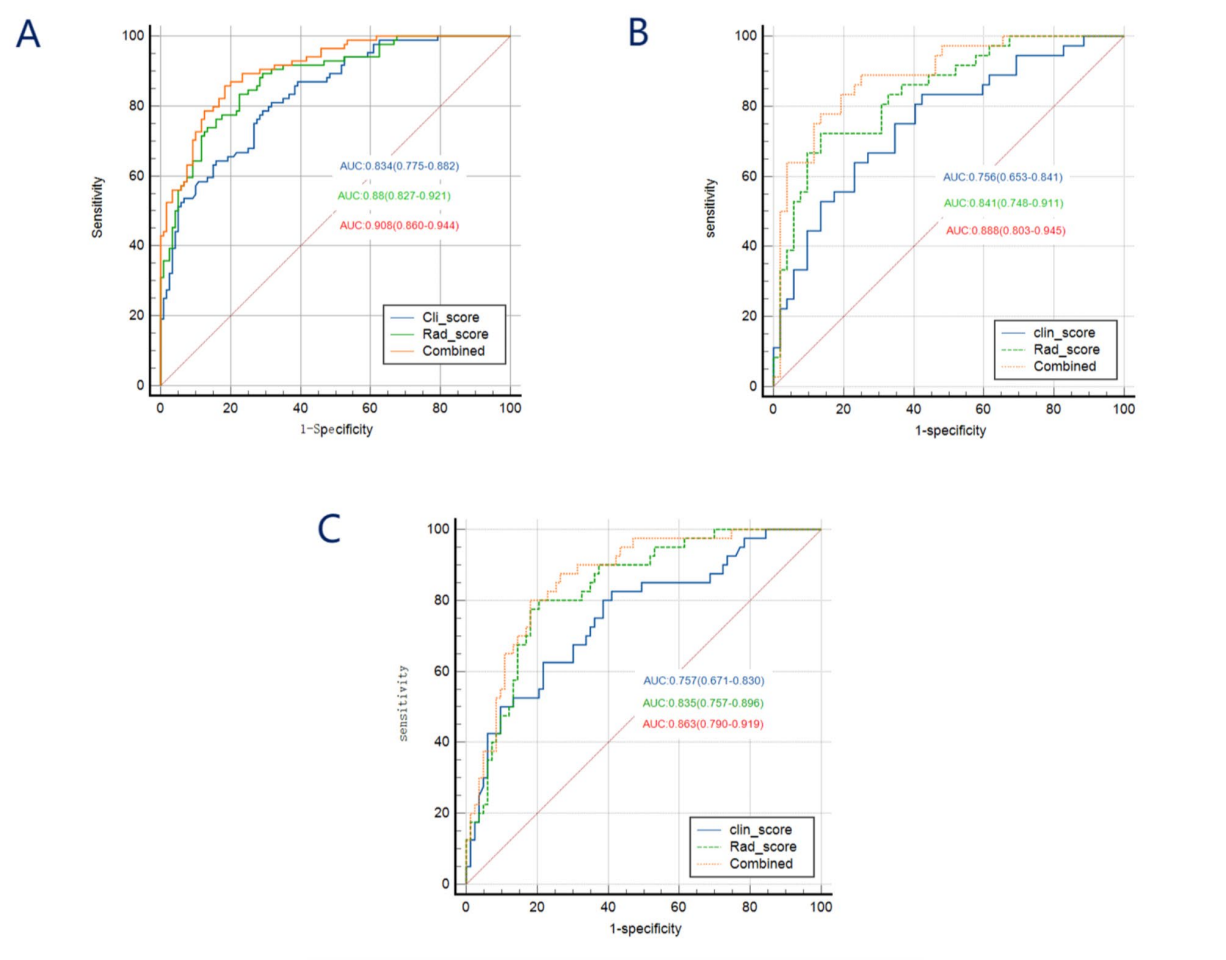
ROC curves and AUC values of different prediction models in the training set (A), validation set (B), and external test set (C). The red, green, and blue colors represent the ROC curves of the combined prediction model, Rad-score model, and Cli-score model predicting the postoperative recurrence of non-functioning pituitary adenoma, respectively.

**Fig. 8:**
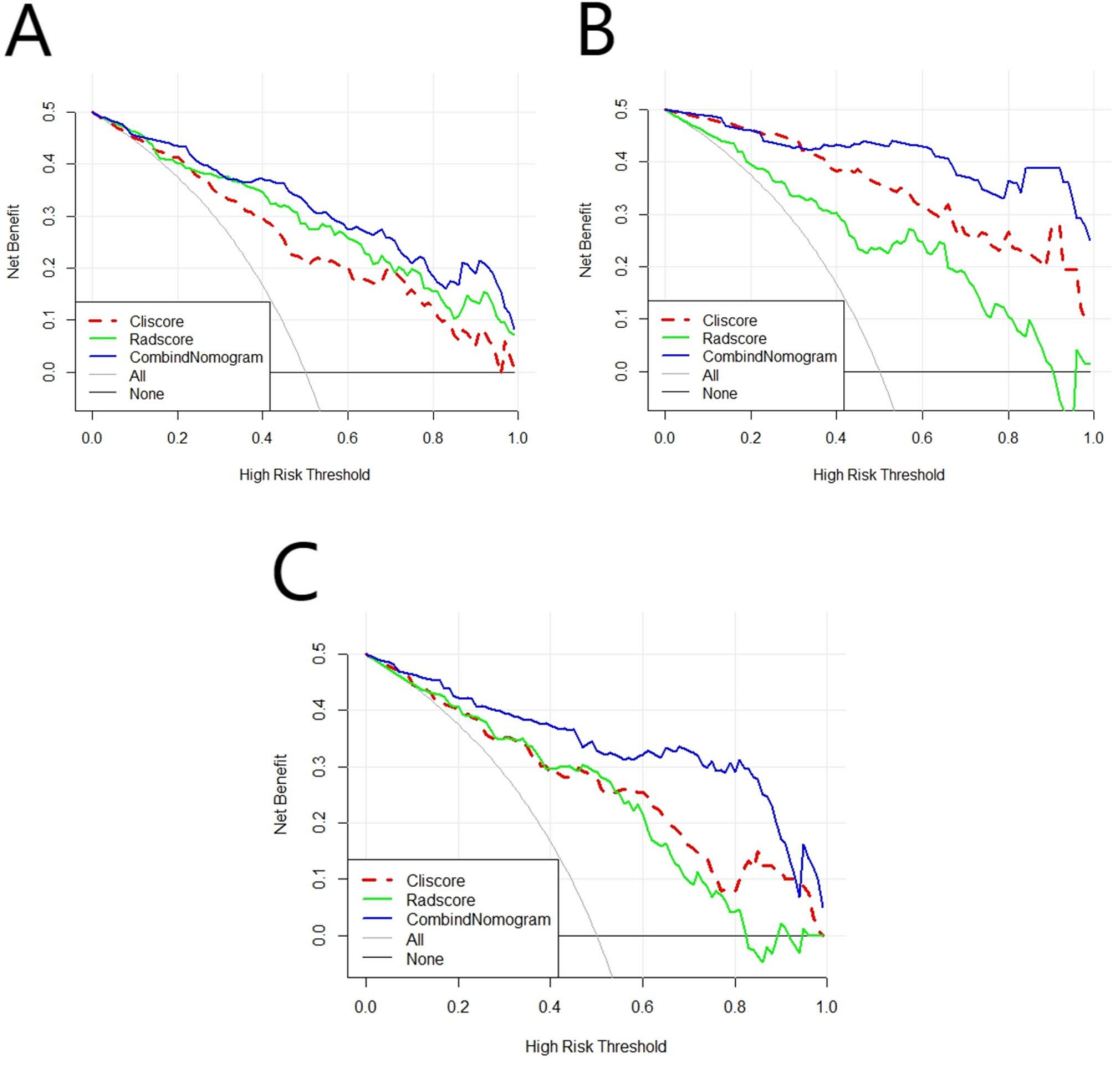
DCA curves of different prediction models in the training set (A), validation set (B), and external test set (C). The blue, green, and red colors represent the combined prediction model, Rad-score model, and Cli-score model, respectively. The X-axis represents the threshold probability, and the Y-axis represents the net benefit.

The ROC curves of the Rad-score model, Cli-score model, and combined model in the training set, validation set, and external test set are shown in Fig. 7. The Delong test showed that in the training set, compared with the clinical model and the radiomics model, the combined prediction model had the highest predictive performance, with statistically significant differences (*P* = 0.0006, *P* = 0.0217); the predictive performance of the radiomics model was higher than that of the clinical model, but the difference was not statistically significant (*P* = 0.1448). In the validation set, the predictive performance of the combined prediction model was also higher than that of the clinical model and the radiomics model, with statistically significant differences (*P* = 0.0118, *P* = 0.0219); the predictive performance of the radiomics model was higher than that of the clinical model, but the difference was not statistically significant (*P* = 0.2028). In the external test set, compared with the clinical model and the radiomics model, the combined prediction model had the highest predictive performance, with statistically significant differences (*P* = 0.0431, *P* = 0.0094); the predictive performance of the radiomics model was higher than that of the clinical model, but the difference was not statistically significant (*P* = 0.1995). The decision curve (Fig. 8) showed that, whether in the training set, validation set, or external test set, within most threshold probability ranges, the net benefit rate of the combined prediction model was higher than that of the Cli-score model and the Rad-score model. Therefore, predicting the recurrence of pituitary adenomas after surgery based on the combined prediction model could provide the greatest clinical benefit to patients.

## Discussion

Postoperative recurrence of pituitary adenomas is characterized by the reappearance of symptoms and signs, and the recurrence of tumor growth on imaging examinations in the pituitary adenoma^19^. Recurrence of non-functional pituitary adenomas primarily relies on imaging examinations for diagnosis, with pituitary-enhanced MRI being the preferred choice^20^. Clinical factors, including the maximum diameter of the tumor before surgery, invasion of the cavernous sinus, and incomplete tumor removal during surgery, are factors influencing tumor recurrence and poor prognosis. Pituitary adenomas with increased proliferative activity and special subtypes have a higher tendency for recurrence^21^. Research by Ko^22^ et al. showed that non-functional pituitary adenomas with invasion of the cavernous sinus, failed decompression of the optic chiasm, large tumor height and volume, low ADC values and its ratio, and high signal on diffusion-weighted imaging are more prone to recurrence. In 2013, Trouillas^23^ et al. formulated a five-tier clinical pathological classification based on radiological, surgical, and pathological elements (1a: non-invasive; 1b: non-invasive but proliferative; 2a: invasive; 2b: invasive and proliferative; 3: metastatic). Comprehensive studies based on radiological, surgical, and pathological factors showed a significant increase in the risk of progression and recurrence in grade 2b (proliferative and invasive) tumors. Research by Lv et al.^24^ demonstrated the significant value of this clinical pathological classification of pituitary tumors in predicting prognosis. Guaraldi et al.^25^ found that Trouillas grade 2, HPF mitosis > 2/10hpf, Ki-67 ≥ 3%, p53 protein expression, tumor infiltration, and ACTH subtype are risk factors for recurrence/progression of pituitary adenoma.

In our study, the results showed that subtotal resection and extensive resection are independent risk factors for recurrence after surgery for non-functional pituitary adenomas (OR = 3.522, *P* = 0.002; OR = 43.685, *P* = 0.001). Previous studies have shown that postoperative residue is the main factor for pituitary tumor recurrence^26^, therefore, more aggressive treatment strategies should be adopted for patients with residual tumors outside the saddle. Other studies have shown that no surgical residue is always associated with a lower recurrence risk^27,28^. These studies are consistent with our research results.

In the 2021 WHO classification of central nervous system tumors, special subtypes of pituitary tumors with more invasive behavior and higher recurrence risk were confirmed^29^. Our research found that the odds of postoperative recurrence in PRL-positive NFMA decreased (OR = 0.226, *P* = 0.038), and other hormone types were not related to postoperative recurrence of NFMA. However, a study by Asioli^30^ et al. reported that PRL, ACHT, and FSH-LH subtypes have a higher risk of recurrence, which is inconsistent with our results. This may be related to differences in case inclusion criteria and sample size. Our study only included patients with non-functional pituitary adenomas, so this needs further research.

The morphology of a tumor is also related to its biological behavior. Generally, tumors with higher malignancy tend to have irregular shapes, often presenting as lobulated, and they exhibit stronger invasiveness to surrounding structures. In contrast, tumors with lower malignancy have more regular shapes, often appearing as rounded, mainly exerting pressure on the surrounding tissues. In our study, the tumor shapes of dumbbell-like (OR = 4.981, *P* = 0.031), irregular (OR = 4.816, *P* = 0.039), and lobulated (OR = 10.446, *P* = 0.022) were identified as independent risk factors for the recurrence of non-functional pituitary adenomas after surgery, and thus, we included them in the predictive model.

Ki-67 is a proliferation marker expressed at different stages of the cell cycle and is the most extensively studied protein in MIB-1 immunohistochemistry. The Ki-67 proliferation index in pituitary tumors is mostly between 1-2%^31^. Almeida reported a higher correlation of Ki-67 > 5% with pituitary tumor recurrence^32^. Currently, the efficacy of Ki-67 as a prognostic factor for pituitary tumors remains controversial. In a review involving 28 Ki-67 studies, 18 reported high expression of Ki-67 in recurrent adenomas, while the other 10 studies showed no correlation^33^. In our study, multivariate logistic regression analysis showed that Ki-67 is an independent risk factor for predicting the postoperative recurrence of non-functioning pituitary adenomas (OR = 3.14, *P* = 0.042), which is consistent with most research results. Therefore, we also included it in the predictive model for the postoperative recurrence of non-functioning pituitary adenomas.

While many factors can influence the postoperative recurrence of pituitary tumors, according to numerous studies, there is no single marker that can independently predict the recurrence trend of pituitary tumors. Therefore, the use of comprehensive models and grading methods, which include various prognostic factors, to predict the prognosis of pituitary tumors has become a trend, assisting clinicians in accurately assessing therapeutic effects and long-term prognosis. Pappy et al.^34^ established a predictive model for predicting persistent high-secreting syndrome and residual adenomas using three important parameters: adenoma diameter, invasion of the cavernous sinus, and Ki-67, demonstrating excellent predictive performance. Lyu et al.^35^ constructed a nomogram to predict the post-operative recurrence of non-functioning pituitary adenomas, which included age, invasion of the cavernous sinus, tumor size, invasion of the sphenoid sinus, and the scope of surgery. The predictive efficacy of this model was high. Additionally, Chen et al.^36^ formulated a prognostic nomogram based on the extent of resection, body mass index, Ki-67, Knosp classification, and smoking status. The results showed that the AUCs for 1-year, 2-year, and 3-year survival periods were 0.889, 0.885, and 0.832, respectively. However, both of these studies were single-center designs and lacked internal and external validation cohorts, which affected the model’s generalizability^37^. Lu et al.^38^ established a predictive model based on pseudocapsular resection, invasion of the cavernous sinus, and tumor size and drew a nomogram, showing good predictive performance. In our study, in addition to patient age, sub-total and gross total resection of the tumor, tumor shape being dumbbell-shaped, irregular, lobulated, PRL subtype, and Ki-67 ≧ 3 were identified as independent risk factors for predicting postoperative recurrence of pituitary tumors. A higher tumor T2 signal intensity ratio was also an independent risk factor. A high T2 signal may indicate more tumor substance, less fibrous stroma, and more active tumor cell proliferation. We used these features to calculate the Cli-score and established a clinical prediction model for predicting the postoperative recurrence of non-functioning pituitary adenomas, with good predictive performance. The DCA curve also indicated that the Cli-score model has high clinical application value. Our research results are similar to the aforementioned studies and are multicenter, further demonstrating the feasibility of using multiple clinical, imaging, and pathological factors to predict the postoperative recurrence of NFMA.

In this study, we applied MR imaging radiomics to predict the postoperative recurrence of non-functioning pituitary adenomas. By reducing the dimensionality of the radiomic features of preoperative T1WI, T2WI, and T1-enhanced images and selecting 14 features related to the postoperative recurrence of non-functioning pituitary adenomas, we calculated the Rad-score based on the logistic regression analysis formula. It exhibited high predictive accuracy in the validation and external test sets. The DCA curve showed that the Rad-score model has high clinical application value. The Delong test indicated that the radiomics predictive model and the clinical prediction model had statistically equivalent predictive performance. Zhang et al.^16^ used a support vector machine (SVM) classifier to evaluate the importance of extracted MR parameters and established a predictive model using the three most important parameters. They found that the radiomics predictive model could predict the postoperative recurrence of non-functioning pituitary adenomas. Machado et al.^39^ also found that the model based on 3D features had an accuracy rate of up to 96.3%, and the model based on 2D features had an accuracy rate of up to 92.6%. However, neither of their studies validated the established models, had small sample sizes, and did not integrate the clinical-imaging-pathological features of patients into the subsequent comprehensive model. Nevertheless, their studies, along with ours, proved the value of the radiomics model in predicting the postoperative recurrence of non-functioning pituitary adenomas.

To further improve our predictive model and enhance the accuracy of predictions, we combined the selected clinical features with the Rad-score to establish a combined predictive nomogram model. Its AUC values in the training set (0.908), validation set (0.888), and external test set (0.863) were higher than those of the standalone Rad-score and Cli-score models. The calibration curve showed good prediction results, and the DCA curve indicated that the combined model has high clinical application value. The Delong test showed that the combined predictive model had statistically significant differences in predictive performance compared to the Rad-score and Cli-score models in the training, validation, and test sets, indicating that the combined predictive model has the best predictive performance. Zhang et al.^40^ used preoperative axial T1-enhanced images of 168 patients to establish two multilayer perceptron (MLP) models to predict the recurrence of pituitary macroadenomas within 5 years, finding that the AUC of the combined model was superior to the clinical-pathological model. Chen et al.^41^ used a deep learning classifier comprising MLP and convolutional neural networks (CNN) to establish a predictive model. They found that the multimodal CNN-MLP model using clinical and MRI features performed best in predicting the recurrence of NFMAs compared to using MRI-only CNN. Both of their studies were consistent with ours and demonstrated that the combined model, established using clinical features combined with radiomic features, had the highest efficacy in predicting the postoperative recurrence of NFMA.

Our study is subject to several limitations that could potentially influence the interpretation and generalizability of our findings. Firstly, the retrospective nature of our investigation introduces the possibility of bias in patient selection and data collection processes. Unlike prospective studies, retrospective analyses are inherently unable to account for all potential confounding variables, which, if left unobserved or unrecorded, might exert an impact on the outcomes of the study. Secondly, our research was confined to non-functioning pituitary macroadenomas (NFMA), thereby excluding both functioning pituitary tumors and microadenomas from our analysis. While this decision was made to mitigate the effects of varying hormone secretion types and to navigate the difficulties associated with precisely outlining the regions of interest (ROIs) for microadenomas, it consequently circumscribes the applicability and broad generalizability of our results to other categories of pituitary tumors.

In contrast to automated deep learning models, the traditional radiomics methodology employed in this study presents certain drawbacks. Manual feature extraction, which is integral to radiomics, can introduce a degree of subjectivity and intricacy into the analysis. In contrast, deep learning models are capable of autonomously discerning features, thereby diminishing the need for human intervention and enhancing the consistency and reproducibility of the findings. Additionally, radiomics features are exceptionally sensitive to fluctuations in a multitude of factors, including but not limited to scanner manufacturers, models, and versions, as well as acquisition protocols and reconstruction settings. Such sensitivities can spawn inconsistencies among features and may compromise the model’s capacity to generalize. Moreover, the majority of radiomics research has been centered on oncological applications, where there is a notable diversity in imaging parameters and scanning protocols across various scanners. The lack of full standardization in analytical software further exacerbates these challenges, significantly curtailing the precision and reproducibility of research outcomes. Consequently, deep learning methodologies, with their promise of greater automation compared to conventional approaches, might be better positioned to advance towards clinical application. However, deep learning models often require a large amount of labeled data for training, which may not be readily available for non-functioning pituitary adenomas. In addition, the training process of deep learning models demands significant computational resources, especially hardware devices such as GPUs or TPUs.

## Conclusion

Both the clinical model constructed using patients’ clinical features and the radiomics model constructed using MR imaging radiomics features can help predict tumor recurrence within 5 years after surgery in NFMA patients. The predictive performance of the combined model, which integrates patients’ clinical features and radiomics features, is superior to that of the standalone clinical model and radiomics model. Furthermore, the model constructed in this study was validated in an external dataset from another hospital. This model will aid in predicting the recurrence within five years after surgery for NFMA, thereby guiding the selection of postoperative follow-up strategies for non-functioning pituitary macroadenoma patients and advising high-risk patients to initiate adjuvant therapy early.

## Data Availability

The datasets used and analysed during the current study available from the corresponding author on reasonable request.

## Abbreviations

NFMA: Non-functioning pituitary macroadenomas
LASSO: Least absolute shrinkage and selection operator
ROC: Receiver operating characteristic curve
AUC: Area under curve
DCA: Decision curve analysis
AIC: Akaike information criterion
OR: Odds ratios
Cli-score: Clinical factor score
DICOM: Digital imaging and communications in medicine
ROI: Region of interest
ICC: Inter-and intra-class correlation coefficient
GLCM: Gray level co-occurrence matrix
GLSZM: Gray-level size zone matrix
GLRLM: Gray-level run-length matrix
NGTDM: Neighborhood gray tone difference matrix
GLDM: Gray-level dependence matrix
LoG: Laplacian of Gaussian
SEN: Sensitivity
SPE: Specificity
ACC: Accuracy
Rad-score: Radiomics score
MLP: Multilayer perceptron
CNN: Neural networks
